# LncRNA MCM3AP-AS1 regulates miR-142-3p/HMGB1 to promote LPS-induced chondrocyte apoptosis

**DOI:** 10.1186/s12891-019-2967-4

**Published:** 2019-12-13

**Authors:** Yanjun Gao, Hongyu Zhao, Yang Li

**Affiliations:** 1 First Department of Orthopedics, The Fourth Affiliated Hospital of China Medical University, Liaoning Province, Shenyang City, 110032 PR China; 2 Comprehensive surgical, Shenyang Orthopedic Hospital, Liaoning Province, Shenyang City, 110044 PR China; 3 Department of Orthopedics, The First People’s Hospital of Shenyang, Liaoning Province, Shenyang City, 110044 PR China

**Keywords:** MCM3AP-AS1, Osteoarthritis, miR-142-3p, Chondrocytes, HMGB1

## Abstract

**Abstract:**

**Background:**

The role of long non-coding RNA (lncRNA) Minichromosome Maintenance Complex Component 3 Associated Protein (MCM3AP) Antisense RNA 1 (MCM3AP-AS1) has been analyzed in liver cancer. But its role in osteoarthritis (OA) is unknown. Through bioinformatics analysis, we predicted that MCM3AP-AS1 may interact with miR-142-3p, which is a major player in OA. This study aimed to investigate the roles of MCM3AP-AS1 in OA and to explore its interactions with microRNA miR-142-3p.

**Methods:**

Differential expressions of MCM3AP-AS1 in OA patients and healthy participants were analyzed by performing quantitative PCR (qPCR). To analyze the relationship between MCM3AP-AS1 and miR-142-3p, human chondrocytes were transfected with MCM3AP-AS1 over-expression vector and miR-142-3p mimic. MCM3AP-AS1, miR-142-3p and high mobility group protein B1 (HMGB1) mRNA expression levels were measured by qPCR.

**Results:**

We found that MCM3AP-AS1 was up-regulated in OA. Bioinformatics analysis showed that MCM3AP-AS1 may interact with miR-142-3p, which can inhibit the apoptosis of chondrocytes. In addition, over-expression of MCM3AP-AS1 and miR-142-3p failed to affect the expression of each other. Instead, MCM3AP-AS1 over-expression led to up-regulated expressions of HMGB1, which is a target of miR-142-3p. Lipopolysaccharide (LPS) treatment led to the up-regulated expressions of MCM3AP-AS1 in chondrocytes. In cell apoptosis assay, MCM3AP-AS1 and HMGB1 over-expression led to increased apoptotic rate of chondrocytes. MiR-142-3p over-expression played an opposite role and attenuated the effects of MCM3AP-AS1 over-expression.

**Conclusions:**

MCM3AP-AS1 may regulate miR-142-3p/HMGB1 to promote LPS-induced chondrocyte apoptosis.

## Background

As the major subtype of arthritis, osteoarthritis (OA) is usually accompanied by cartilage degeneration [1]. The development of OA damages joints and causes chronic pain and disability [2]. Although it is not lethal, the direct medical cost and the indirect productivity loss pose heavy economic burden on the modern society [3]. OA affects about 15% of populations over 18 years’ old [4]. Aging is the main risk factor for OA. Occurrence of OA is predicted to be furtherly increased in near future, owing to the growing of aged population [5]. Treatment of OA is mainly focused on pain relief and disease control [6]. However, effective disease therapeutic approaches remain to be elusive.

Besides the well-characterized risk factors such as aging and obesity, genetic factors are also major participants in the development of OA [7]. It is predicted that the identification of novel genetic factors in OA may help to develop targeted therapies to recover the pathological changes [8]. Non-coding RNAs (ncRNAs), such as miRNAs, are frequently dysregulated during the development of OA [9]. Some miRNAs, such as miR-142-3p, may improve the conditions of OA by inhibiting cell apoptosis and inflammation by targeting high mobility group protein B1 (HMGB1) [10]. However, the upstream regulation of miR-142-3p/HMGB1 in OA is unclear. MCM3AP Antisense RNA 1 (MCM3AP-AS1) is a recently characterized oncogenic long (> 200 nt) ncRNA (lncRNA) in hepatocellular carcinoma [11]. Through bioinformatics analysis, we predicted that MCM3AP-AS1 may interact with miR-142-3p. This study aimed to investigate the interaction between MCM3AP-AS1 and miR-142-3p in OA.

## Methods

### OA patients and healthy participants

The Fourth Affiliated Hospital of China Medical University Ethics Committee approved this study (No. FAHCMU20160245298, the investigation of lncRNA MCM3AP-AS1 in LPS-induced chondrocyte apoptosis). Research subjects of the present study were 70 cases of OA patients (stage III, 30 cases; stage IV, 40 cases; 26 males and 44 females; age range from 54 to 72 years’ old; mean age at 63.1 ± 6.0 years) and 70 healthy volunteers (26 males and 44 females, age range from 54 to 72 years’ old; mean age at 63.0 ± 6.1 years’ old), who were enrolled at aforementioned hospital between May 2016 and May 2019. Inclusion criteria of OA patients were the following: 1) no therapies performed within 100 days; 2) newly diagnosed OA. Exclusion criteria: 1) OA patients complicated with other clinical disorders; 2) recurrent OA. The OA patients were diagnosed by conventional techniques, such as joint fluid analysis and X-ray. The 70 healthy participants received routine physical examinations at the physical health center of aforementioned hospital. Among the 70 OA patients, lesions in knee were observed in 39 cases and lesions in hip were observed in remaining 31 cases. They were selected to match OA patients’ age and gender distributions. All OA patients (*n* = 70) and healthy participants (n = 70) were informed of experimental principle and informed consent was provided by all the 140 participants.

### Synovial fluid

Synovial fluid (2 ml) was extracted from the affected sites of patients by performing arthrocentesis. To match the conditions of the OA group, synovial fluid extraction from knee was performed on 39 cases of healthy participants and extraction from hip was performed in 31 cases of healthy participants.

### Primary chondrocytes and transient transfections

Primary chondrocytes (obtained from adult OA patients) from Sigma-Aldrich (402OA-05A, St. Louis, MO, USA) were used. Cells were cultivated in a 5% CO_2_ incubator at 37 °C with 95% humidity. The cell culture medium was Chondrocyte Growth Medium from PromoCell (Heidelberg, Germany). Following transfection experiments were performed using cells from passage 4–6. At passage 4–6, no significant changes in viability and morphology were observed.

The expression vector of MCM3AP-AS1 or HMGB1 was constructed using pcDNA3.1 vector as backbone. The vector construction service was provided by Genecopoeia (Guangzhou, China). Negative control (NC) miRNA and miR-142-3p mimic were synthesized by Beyotime Biotechnology (Shanghai, China). Transient transfections were mediated by lipofectamine™ 2000 (Invitrogen, Carlsbad, CA, USA). Chondrocytes (10^6^) were transfected with 40 nM miRNA (NC miRNA as NC group) or 10 nM expression vector (empty vector as NC group) through the methods described by Invitrogen. In all cases, untransfected cells were control (C) cells. Subsequent experiments were performed using cells harvested at 24 h after transfections.

### RNA-RNA interaction prediction

The interaction between MCM3AP-AS1 and miR-142-3p was predicted using an online program named IntaRNA (http://rna.informatik.uni-freiburg.de/IntaRNA/Input.jsp) [12]. MCM3AP-AS1 was used as the long sequence and miR-142-3p was the short sequence. Other parameters are the default.

### Dual-luciferase reporter assay

psiCHECK-1 vector (Promega, Chicago, IL, USA) was used to construct the MCM3AP-AS1 expression vector. MCM3AP-AS1 expression vector combined with NC miRNA (NC group) or miR-142-3p mimic (miR-142-3p group) was transfected into 10^5^ chondrocytes using aforementioned methods. Luciferase Reporter Assay Kit I (Firefly, PromoCell) was used to measure luciferase activity. Relative luciferase activity was calculated using firefly luminescence according to manufacturer’s instructions.

### RNA extractions and digestion

TRIzol from Thermo Fisher Scientific (Cincinnati, OH, USA) was used to perform RNA extractions from both synovial fluid and chondrocytes. MiRNAs were harvested by precipitating RNAs using 85% ethanol. Genomic DNAs were removed by digesting RNA samples using gDNA Eraser (TaKaRa, Tokyo, Japan). In cases of lipopolysaccharide (LPS) treatment, chondrocytes were cultivated in medium containing 0, 500, 1000 and 2000 ng/ml LPS (Sigma-Aldrich) for 24 h under aforementioned methods to mimic OA conditions before use.

### RT-PCR

SSRT IV system (Thermo Fisher Scientific) was used to reverse transcribe total RNAs into cDNAs with poly (T) as primer. To measure expression levels of MCM3AP-AS1 and HMGB1 mRNA, SensiFAST™ Real-Time PCR Kit (Bioline, Memphis, TN, USA) was used to perform quantitative PCR (qPCR) assays with Glyceraldehyde-3-Phosphate Dehydrogenase (GAPDH) as endogenous control.

Expression levels of mature miR-142-3p were measured through the following steps: 1) addition of poly (A); 2) reverse transcription; 3) qPCR assays. All steps were performed using the All-in-OneTM miRNA qRT-PCR Detection Kit from GeneCopoeia. Ct values were determined using default threshold and 2^−ΔΔCT^ method [13] was used for all data normalizations.

### Western blot

At 24 h post-transfection, 10^5^ chondrocytes were lysed using RIPA solution (Beyotime, Shanghai, China) to extract total proteins. The concentration of protein in each sample was measured by performing a BCA assay (Beyotime). Following denaturation in boiling water for 15 min, 10% SDS-PAGE gel was used to separate different proteins. Following gel transfer to (NC) membrane, PBS containing 5% fat-free milk was used to block membranes for 2 h at room temperature (RT). Then, primary antibodies including rabbit anti-GAPDH (ab38168, Abcam, London, UK) and anti-HMGB1 (ab18256, Abcam) were used to incubate with the membranes for 15 h at 4 °C, followed by incubating with HRP (IgG) secondary antibody (Goat Anti-Rabbit, ab6721, Abcam) for 2 h at RT. Western-Ready ECL Substrate Kit (BioLegend, San Diego, CA, USA) was used for signal production and ImageJ v.148 software was used for data normalization.

### Cell apoptosis analysis

Cell apoptosis analysis after transfection was performed using flow cytometry. A 6-well cell culture plate was used to cultivate chondrocyte suspension with 2 ml (10^5^ cells). Cell culture medium contained 2000 ng/ml LPS (Sigma-Aldrich). Cells were cultivated under aforementioned methods for 48 h and were trypsinized. After that, Annexin V-FITC (Thermo Fisher Scientific) and propidium iodide (PI, Thermo Fisher Scientific) staining was performed in dark for 20 min and apoptotic cells were detected by flow cytometry using BD Accuri™ C6 Plus Flow Cytometer (BD Biosciences, San Jose, CA, USA).

### Statistical analysis

Data from 3 independent biological replicates of each experiment were used to calculate mean values, which were used in the following data analysis. The unpaired t-test was used for data comparison between two groups. Data comparisons among multiple groups were performed by ANOVA (one-way) and Tukey test. *p* < 0.05 was statistically significant.

## Results

### MCM3AP-AS1 was up-regulated in OA

**Fig. 1:**
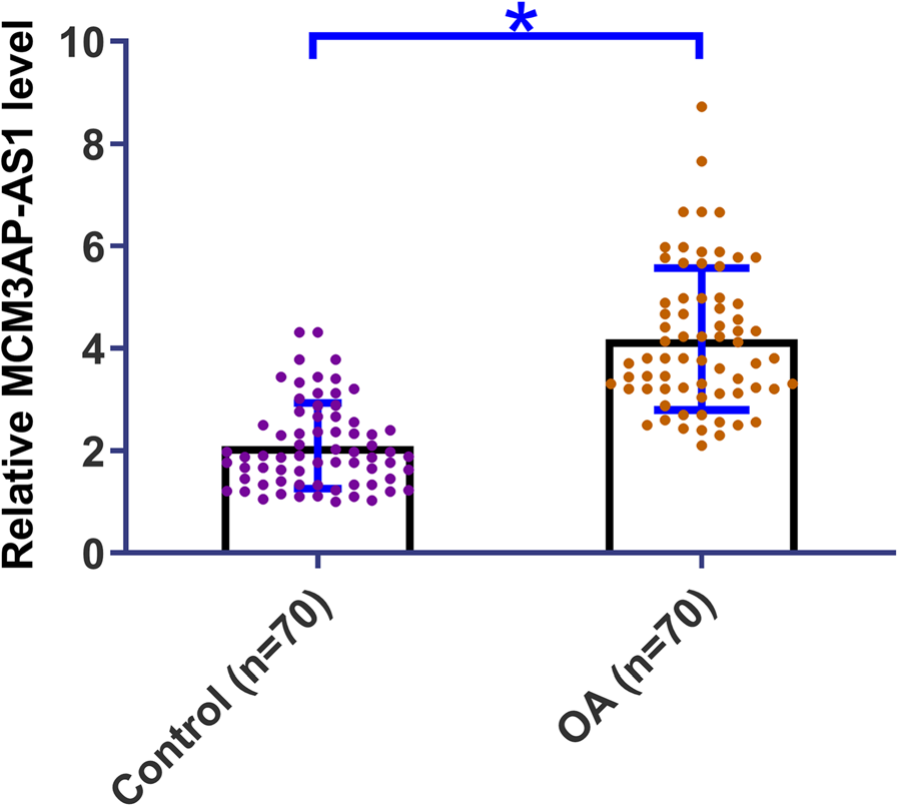
MCM3AP-AS1 was up-regulated in OA. Differential expression of MCM3AP-AS1 in OA patients and healthy participants were analyzed by performing qPCR. A comparison of the expression level of MCM3AP-AS1 between OA group (*n* = 70) and Control group (n = 70) was performed by performing an unpaired t-test. PCR reactions were repeated 3 times and data were expressed as mean values, *, *p* < 0.05

Expression levels of MCM3AP-AS1 in OA patients and healthy participants were measured by performing qPCR. The sample with the lowest expression was set to “1”, and all other samples were normalized to this sample to calculate relative expression levels. A comparison of the expression level of MCM3AP-AS1 between OA group (*n* = 70) and Control group (n = 70) was performed by performing an unpaired t-test. It was observed that expression levels of MCM3AP-AS1 were significantly higher in OA patients than in Control group (1.96-fold, Fig. 1, *p* = 0.02).

### MCM3AP-AS1 can directly interact with miR-142-3p

**Fig. 2:**
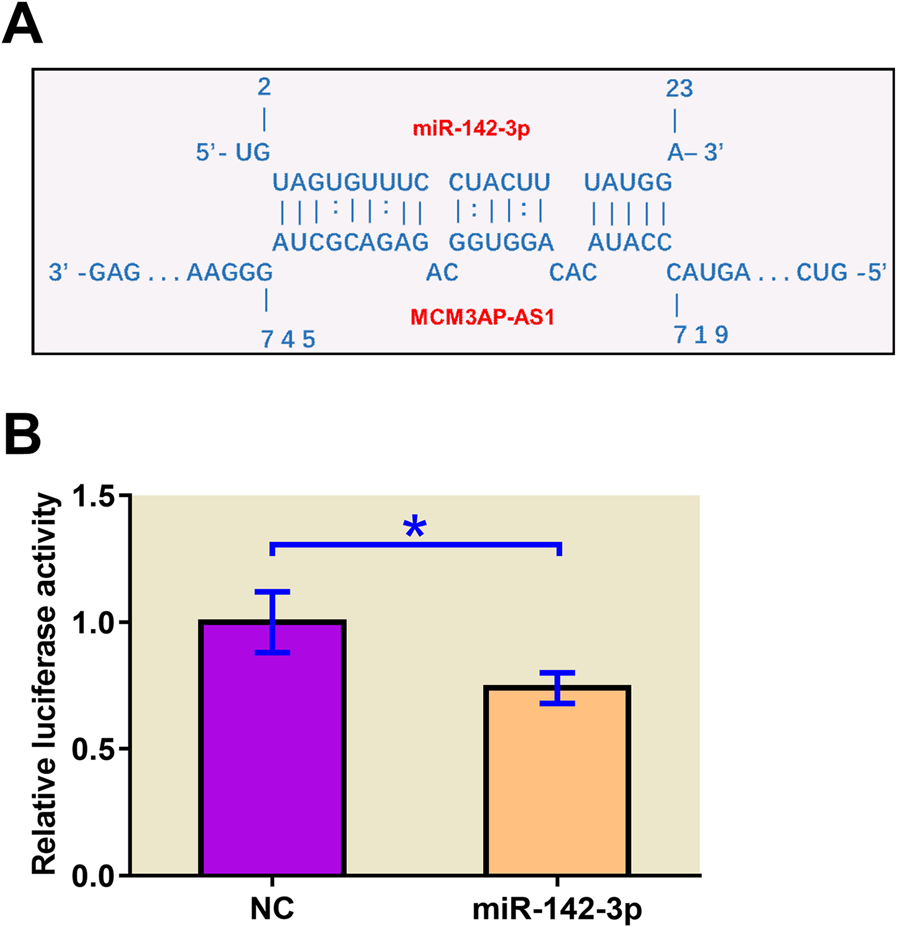
MCM3AP-AS1 can directly interact with miR-142-3p. Bioinformatics analysis performed using IntaRNA showed that MCM3AP-AS1 may interact with miR-142-3p (**a**). Dual-luciferase assay was performed to further analyze the interaction between MCM3AP-AS1 and miR-142-3p (**b**). Experiments were repeated 3 times and data were expressed as mean values. *, *p* < 0.05

IntaRNA was used to analyze the potential interaction between MCM3AP-AS1 and miR-142-3p. It can be observed that MCM3AP-AS1 may interact with miR-142-3p. The free energy was − 16.77 kcal/mol, which indicates strong interaction between them (Fig. 2a). The interaction between MCM3AP-AS1 and miR-142-3p was further analyzed by luciferase assay. Comparing to chondrocytes transfected with MCM3AP-AS1 and NC miRNA, chondrocytes transfected with MCM3AP-AS1 and miR-142-3p showed significantly reduced relative luciferase activity, indicating that miR-142-3p can bind MCM3AP-AS1 to reduce the luciferase activity (Fig. 2b, *p* < 0.05).

### Over-expression of MCM3AP-AS1 and miR-142-3p failed to affect each other

**Fig. 3:**
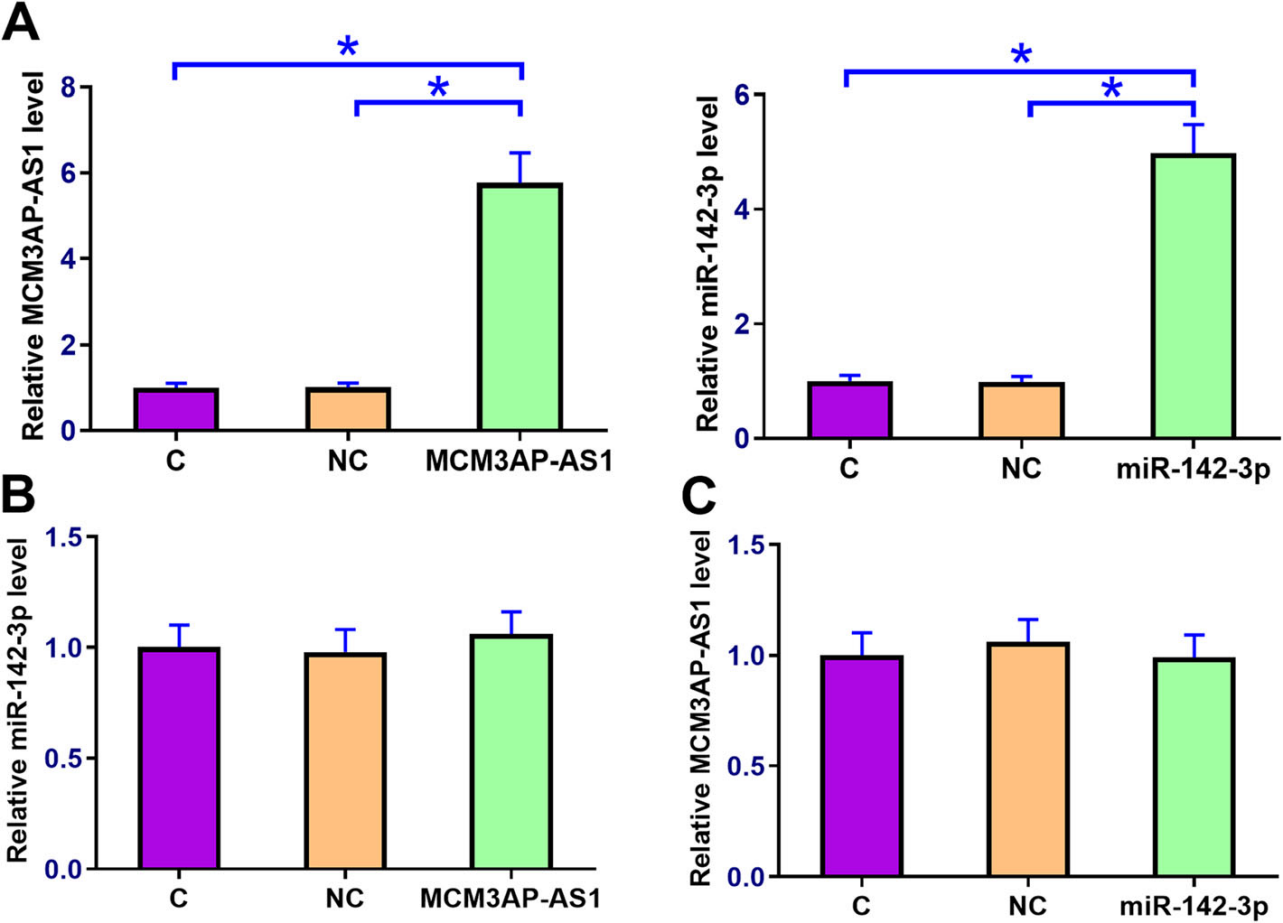
Over-expression of MCM3AP-AS1 and miR-142-3p failed to affect each other. To analyze the relationship between MCM3AP-AS1 and miR-142-3p, chondrocytes were transfected with the MCM3AP-AS1 expression vector and miR-142-3p mimic. Over-expression of MCM3AP-AS1 and miR-142-3p was confirmed by qPCR at 24 h post-transfection (**a**). The effects of MCM3AP-AS1 over-expression on miR-142-3p (**b**) as well as the effects of miR-142-3p over-expression on MCM3AP-AS1 expression (**c**) were analyzed by qPCR. Experiments were repeated 3 times and data were expressed as mean values. *, *p* < 0.05

Chondrocytes were transfected with MCM3AP-AS1 expression vector or miR-142-3p mimic to analyze the interactions between MCM3AP-AS1 and miR-142-3p. qPCR was performed at 24 h post-transfection to confirm the over-expression of MCM3AP-AS1 and miR-142-3p (Fig. 3a, p < 0.05). Control (C) was set to “1” and all other groups were normalized to C group to calculate relative expression levels. Comparing to NC and C groups, cells with MCM3AP-AS1 over-expression showed no significantly altered miR-142-3p expression (Fig. 3b). Similarly, miR-142-3p over-expression failed to significantly affect MCM3AP-AS1 expression (Fig. 3c).

### MCM3AP-AS1 over-expression led to up-regulated HMGB1

**Fig. 4:**
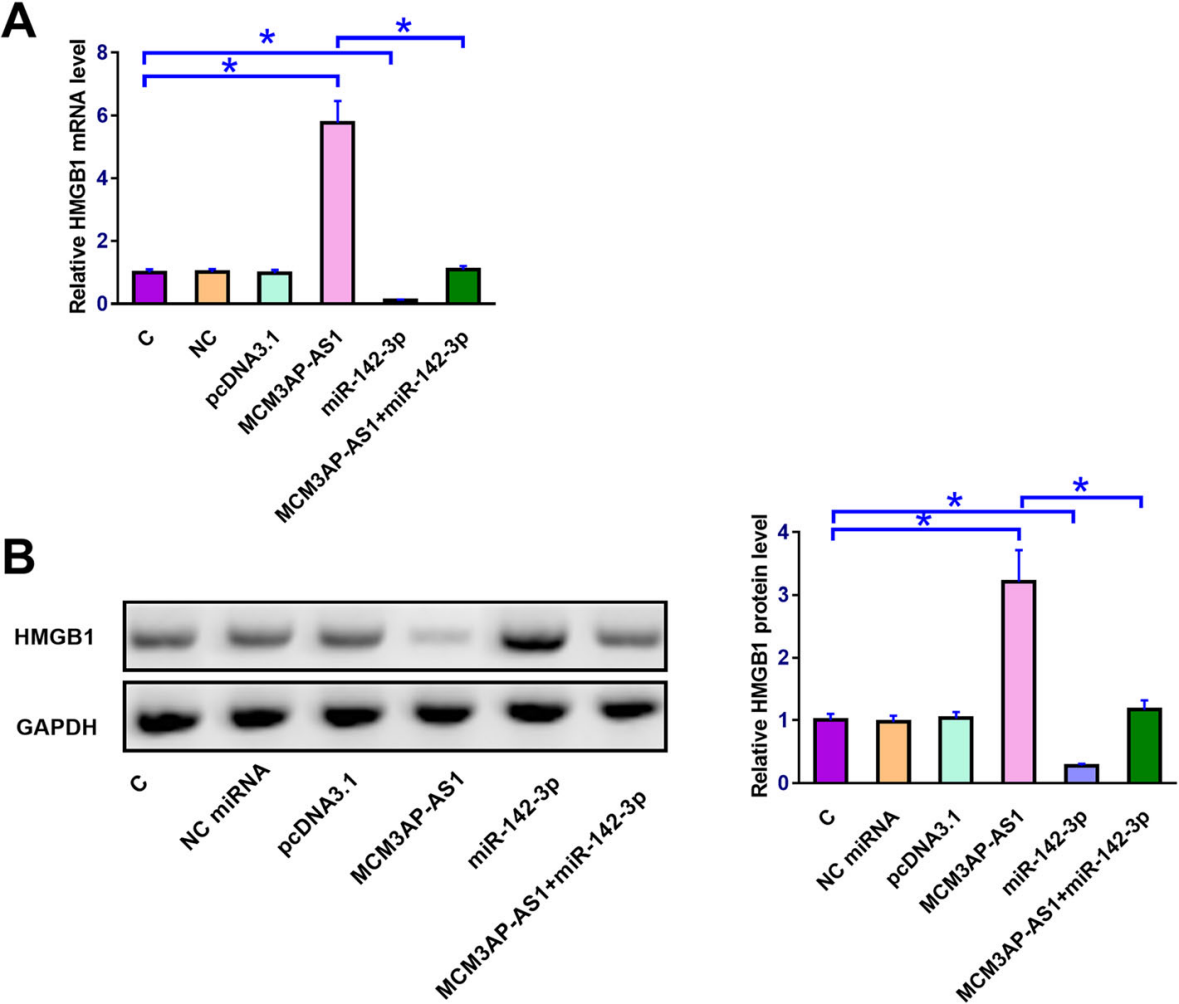
MCM3AP-AS1 over-expression led to up-regulated HMGB1. HMGB1 is a direct downstream target of miR-142-3p. The effects of MCM3AP-AS1 and miR-142-3p over-expression on HMGB1 expression at mRNA and protein levels were analyzed by qPCR (**a**) and western blot (**b**), respectively. Gene names on X-axis indicated the over-expression of this gene. C on X-axis, control cells, cells without transfection, NC on X-axis, negative control, cells transfected with empty vector or NC miRNA. Experiments were repeated 3 times and data were expressed as mean values. *, p < 0.05

HMGB1 expression in chondrocytes with MCM3AP-AS1 and miR-142-3p over-expression at mRNA and protein levels were analyzed by qPCR (Fig. 4a) and western blot (Fig. 4b), respectively. Control (C) was set to “1” and all other groups were normalized to C group to calculate relative expression levels. In chondrocytes, down-regulated HMGB1 expression was observed after miR-142-3p over-expression. In contrast, MCM3AP-AS1 over-expression led to up-regulated HMGB1 and reduced effects of miR-142-3p over-expression.

### MCM3AP-AS1 over-expression promoted LPS-included chondrocyte apoptosis through HMGB1 and miR-142-3p

**Fig. 5:**
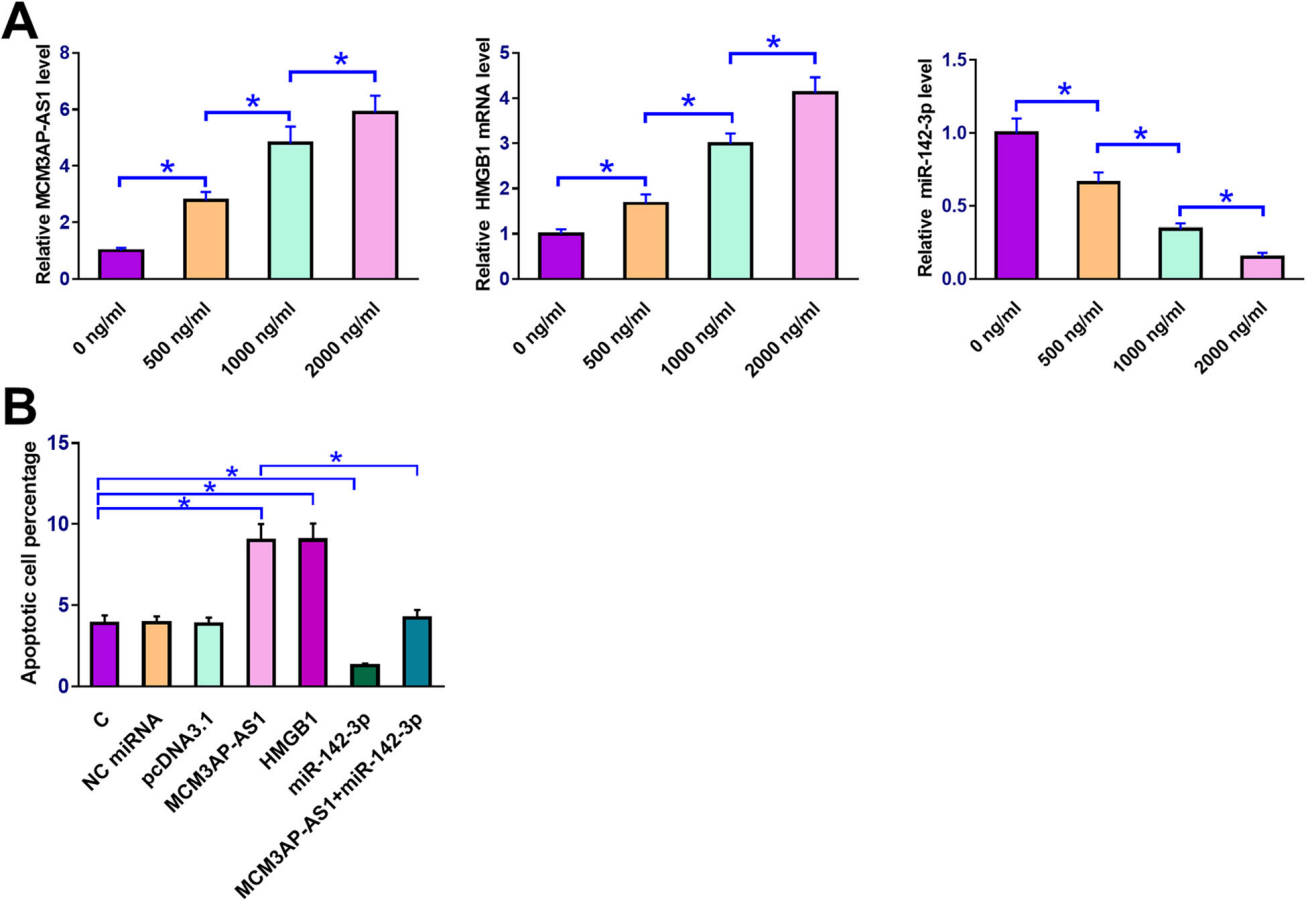
MCM3AP-AS1 over-expression promoted LPS-included chondrocyte apoptosis through HMGB1 and miR-142-3p. Chondrocytes were cultivated in medium containing 0, 500, 1000 and 2000 ng/ml LPS for 24 h under the aforementioned methods, followed by the measurement of MCM3AP-AS1, miR-142-3p and HMGB1 mRNA expression levels by qPCR (**a**). Cell apoptosis assay was performed to analyze the effects of MCM3AP-AS1, miR-142-3p and HMGB1 over-expression on the apoptosis of chondrocytes under 2000 ng/ml LPS treatment (**b**). Gene names on X-axis indicated the over-expression of this gene. **c** on X-axis, control cells, cells without transfection, NC on X-axis, negative control, cells transfected with empty vector or NC miRNA. Experiments were repeated 3 times and data were expressed as mean values. *, p < 0.05

QPCR was performed to measure the expression levels of MCM3AP-AS1, miR-142-3p and HMGB1 mRNA in chondrocytes collected after cell culture in medium containing 0, 500, 1000 and 2000 ng/ml LPS for 24 h. Expression level of corresponding gene in cells treated with 0 ng/ml LPS was set to “1” and all other groups were normalized to this group. It was observed that LPS treatment led to the up-regulated MCM3AP-AS1 and HMGB1 mRNA but down-regulated miR-142-3p in chondrocytes in a dose-dependent manner (Fig. 5a, *p* < 0.05). Cell apoptosis assay showed that compared to C and NC (NC miRNA or pcDNA3.1 vector transfection), MCM3AP-AS1 and HMGB1 over-expression led to increased apoptotic rate of chondrocytes. MiR-142-3p over-expression played an opposite role and attenuated the effects of MCM3AP-AS1 over-expression (Fig. 5b, p < 0.05).

## Discussion

In this study, we found a lncRNA named MCM3AP-AS1 with critical functions in OA. We showed that MCM3AP-AS1 was up-regulated in OA and may promote chondrocyte apoptosis through the regulation of miR-142-3p/HMGB1 axis.

Previous studies have characterized many differentially expressed lncRNAs in OA [14–16]. However, most of the differentially expressed lncRNAs may have no critical functions in disease progression [14, 15]. Some lncRNAs have been reported to play critical roles in OA. For instance, plasmacytoma variant translocation 1 (PVT1) is over-expressed in OA and participates in OA progression by promoting the apoptosis of chondrocytes [17], which are the only cells in cartilage and have critical roles in the maintenance of cartilaginous matrix [18]. In another study, Su et al. reported that maternally expressed 3 (MEG3) is down-regulated in OA and may regulate vascular endothelial growth factor (VEGF) to participate in angiogenesis of OA [19]. The expression and function of MCM3AP-AS1 have only been reported in hepatocellular carcinoma (HCC). In HCC, MCM3AP-AS1 is up-regulated and regulates miR-194-5p/forkhead box protein A1 (FOXA1) axis to promote tumor growth [11]. In this study we first reported the up-regulation of MCM3AP-AS1 in OA. In addition, we also observed increased apoptotic rate of chondrocytes under LPS treatment after MCM3AP-AS1 over-expression. Therefore, MCM3AP-AS1 may promote chondrocyte apoptosis to participate in OA.

In a recent study Wang et al. reported that miR-142-3p targeted HMGB1 to inhibit the apoptosis of chondrocytes [10]. This study basically confirmed the finding of Wang et al. through over-expression experiments. It is known that the functions of miR-142-3p can be regulated by certain lncRNAs [20]. In this study we speculate that MCM3AP-AS1 may be an endogenous sponge of miR-142-3p. This speculation is based on following observations: 1) MCM3AP-AS1 can directly interact with miR-142-3p; 2) over-expression of MCM3AP-AS1 and miR-142-3p failed to affect the expression of each other; 3) MCM3AP-AS1 over-expression led to the up-regulated miR-142-3p target HMGB1. Therefore, this study characterized a novel MCM3AP-AS1/miR-142-3p/HMGB1 pathway involved in OA. However, further experiments, such as in vivo animal model experiments are needed to further explore the mechanism.

## Conclusion

In conclusion, MCM3AP-AS1 was up-regulated in OA and may sponge miR-142-3p to up-regulate HMGB1, thereby promoting LPS-induced chondrocyte apoptosis.

## Data Availability

The analyzed data sets generated during the study are available from the corresponding author on reasonable request.

## Abbreviations

HMGB1: High mobility group protein B1
lncRNA: Long non-coding RNA
MCM3AP: Maintenance Complex Component 3 Associated Protein (MCM3AP) Antisense RNA 1
MCM3AP: Minichromosome Maintenance Complex Component 3 Associated Protein
ncRNAs: Non-coding RNAs
OA: Osteoarthritis

## References

[1] Glyn-Jones S (2015). Palmer A J R, Agricola R, et al. Osteoarthritis Lancet.

[2] Allen KD, Golightly YM (2015). Epidemiology of osteoarthritis: state of the evidence. Curr Opin Rheumatol.

[3] Xie F, Kovic B, Jin X (2016). Economic and humanistic burden of osteoarthritis: a systematic review of large sample studies. Pharmacoeconomics..

[4] Plotnikoff R, Karunamuni N, Lytvyak E (2015). Osteoarthritis prevalence and modifiable factors: a population study. BMC Public Health.

[5] Rahmati M, Nalesso G, Mobasheri A (2017). Aging and osteoarthritis: central role of the extracellular matrix. Ageing Res Rev.

[6] Sinusas K (2012). Osteoarthritis: diagnosis and treatment. Am Fam Physician.

[7] Sandell LJ (2012). Etiology of osteoarthritis: genetics and synovial joint development. Nat Rev Rheumatol.

[8] Mobasheri A (2013). The future of osteoarthritis therapeutics: targeted pharmacological therapy. Curr Rheumatol Rep.

[9] Beyer Christian, Zampetaki Anna, Lin Neng-Yu, Kleyer Arnd, Perricone Carlo, Iagnocco Annamaria, Distler Alfiya, Langley Sarah R, Gelse Kolja, Sesselmann Stefan, Lorenzini Rolando, Niemeier Andreas, Swoboda Bernd, Distler Jörg H W, Santer Peter, Egger Georg, Willeit Johann, Mayr Manuel, Schett Georg, Kiechl Stefan (2014). Signature of circulating microRNAs in osteoarthritis. Annals of the Rheumatic Diseases.

[10] Wang X, Guo Y, Wang C (2016). MicroRNA-142-3p inhibits chondrocyte apoptosis and inflammation in osteoarthritis by targeting HMGB1. Inflammation..

[11] Wang Y, Yang L, Chen T (2019). A novel lncRNA MCM3AP-AS1 promotes the growth of hepatocellular carcinoma by targeting miR-194-5p/FOXA1 axis. Mol Cancer.

[12] Mann M, Wright PR, Backofen R (2017). IntaRNA 2.0: enhanced and customizable prediction of RNA–RNA interactions. Nucleic Acids Res.

[13] Livak KJ, Schmittgen TD (2001). Analysis of relative gene expression data using real-time quantitative PCR and the 2− ΔΔCT method. Methods..

[14] Xing D, Liang J, Li Y (2014). Identification of long noncoding RNA associated with osteoarthritis in humans. Orthop Surg.

[15] Fu M, Huang G, Zhang Z (2015). Expression profile of long noncoding RNAs in cartilage from knee osteoarthritis patients. Osteoarthr Cartil.

[16] Ajekigbe B, Cheung K, Xu Y (2019). Identification of long non-coding RNAs expressed in knee and hip osteoarthritic cartilage. Osteoarthr Cartil.

[17] Li Y, Li S, Luo Y (2017). LncRNA PVT1 regulates chondrocyte apoptosis in osteoarthritis by acting as a sponge for miR-488-3p. DNA Cell Biol.

[18] Archer CW, Francis-West P (2003). The chondrocyte. Int J Biochem Cell Biol.

[19] Su W, Xie W, Shang Q (2015). The long noncoding RNA MEG3 is downregulated and inversely associated with VEGF levels in osteoarthritis. Biomed Res Int.

[20] Li Y, Lv M, Song Z (2018). Long non-coding RNA NNT-AS1 affects progression of breast cancer through miR-142-3p/ZEB1 axis. Biomed Pharmacother.

